# Conceptual relatedness promotes memory generalization at the cost of detailed recollection

**DOI:** 10.1038/s41598-023-40803-4

**Published:** 2023-09-20

**Authors:** Greta Melega, Signy Sheldon

**Affiliations:** 1 Department of Neurology, Charité Universitätsmedizin Berlin, Berlin, Germany; 2 Department of Psychology, McGill University, 2001 McGill College, Montreal, QC H3A 1G1 Canada

**Keywords:** Psychology, Human behaviour

## Abstract

An adaptive memory system is one that allows us to both retrieve detailed memories as well as generalize knowledge about our past, the latter termed memory generalization and is useful for making inferences about new situations. Research has indicated that memory generalization relies on forming knowledge structures by integrating experiences with shared encountered elements. Whether memory generalization occurs more readily when  experiences also have elements that share established (conceptual) information is less clear. It is also unclear if engaging in memory generalization during learning comes at the cost of retrieving detailed memories, the other function of episodic memory. To address these two knowledge gaps, we paired a modified version of the acquired equivalence task with a recognition memory test. Across three experiments, participants first learned a series of overlapping object-scene pairs (A–X, B–X and A–Y) in which half of the overlapping pairs contained conceptually-related objects (e.g., A—pencil; B—scissors; conceptual condition) and the other half contained unrelated objects (neutral condition). Participants ability to generalize to new overlapping object-scene pairs (B–Y) as well as not-learned but semantically-related objects was measured. Finally, participants completed a recognition memory test that included the encoded objects, perceptually similar lures or new foil objects. Across all experiments, we found higher rates of generalization but reduced detailed memory (indexed by increased false alarms to lure objects) for information learned in the conceptual than neutral condition. These results suggest the presence of conceptual knowledge biases an individual towards a generalization function of memory, which comes at the expense of detailed recollection.

## Introduction

An adaptive memory system is one that allows us to both remember specific details from an individual episode, such as a recollecting that last night you left the keys in your pocket instead of placing them next to the front door, as well as form a general representation of such events to make inferences about new experiences, such as considering that your flatmate would normally place their keys next to the door as you have done several times before. A wealth of research has documented that episodic memory is critical for retrieving detailed experiences^1^ and more recent work has found that episodic memory is also necessary for forming general memory representations^2,3^. According to this work, episodic memory engages memory generalization processes to integrate experiences with shared elements into a general knowledge structure that can then be applied to make inferences about new situations^4–7^. Several questions remain about how generalization occurs. In the current study, we aimed to address two questions related to the role of conceptual (semantic) knowledge in memory generalization. First, given the established role of conceptual knowledge in episodic memory processing, we asked whether generalization occurs more readily when experiences share conceptual elements. Second, given the role of episodic memory in both generalization and detailed memory, we asked whether engaging in generalization comes at the cost of detailed recollection for learned information. To answer our study questions, we modified a well-validated measure of memory generalization, the acquired equivalence task, and paired it with a recognition memory test.

## Memory generalization measured through acquired equivalence and the link to conceptual knowledge

Acquired equivalence occurs when an individual first learns to associate a pair of items that share a common link, such as learning that both a mango tree and a papaya tree are located in Hawaii. Then, when the individual learns new information about one of the item pairs, such as learning that papaya trees are also located in India, they infer that this new information also pertains to the related item pair, such as assuming mango trees are also located in India. Thus, a common paradigm to test for acquired equivalence will measure how information encoded within learned pairs (papaya tree—Hawaii, mango tree—Hawaii, and papaya tree—India) generalizes to indirectly related pairs, those that share common elements across learned pairs (mango tree—India)^8,9^. Experiments using this paradigm have illustrated that generalization does occur across these types of learned experiences, with many interpreting the results of these experiments as indicating that generalization results from integrating common elements of experiences during encoding^3,4^. Yet, such integration—and generalization—might also occur by relating newly learned information from an experience to another that shares common prior conceptual knowledge (e.g., inferring that mango and papaya trees are both present in India and Hawaii as most exotic fruits grow at similar latitudes).

The notion that generalization might also be based on integrating information that shares conceptual information is based on several studies indicating that prior knowledge, our storehouse of conceptual information, has a strong influence on several functions of episodic memory (see^10^ for a review). For example, several studies have shown that episodic memory retrieval is facilitated by the presence of pre-existing semantic knowledge^11–15^ and is enhanced in people with greater prior knowledge of a particular concept^16^. In terms of memory generalization, recent work has hinted that the presence of conceptually related information boosts generalization^17^. For example, prior work asked participants to identify similarities across single episodes (e.g., between A–B pairs and A–C pairs) and then make decisions about new but indirectly learned pairs (B–C pairs). The results were that when elements B and C were semantically congruent (conceptually related) within a learning scenario (e.g., rubber duck and bathroom), there were high rates of memory integration and generalization^18,19^. Yet, in these studies, conceptual relatedness was dependent on the learning scenario, and so the question remains whether conceptual generalization occurs when elements share a common concept drawn from prior knowledge and not entangled within a learning experience^20^. In other words, do we naturally include conceptual relations when integrating information into the knowledge structure that guides generalization?

## The relationship between memory generalization and detailed memory

As previously mentioned, two functions of episodic memory are the ability to form detailed memories as well as general representations that are based on commonalities across multiple experiences. There are hints from the literature that engaging in memory generalization might dampen this other function of episodic memory, the ability to form detailed memories^21,22^. These hints come from a collection of studies that have found that promoting memory generalization when encoding information leads to an inaccurate detailed recollection of that information, such as a greater number of source misattributions from where that information was learned^21–23^. As one specific example, an experiment was conducted in which participants encoded overlapping pairs of items (A–B, B–C) where one element (A) was rewarded. To measure generalization, the researchers tested whether participants would attribute the reward to an indirectly associated element (C). They found that participants who successfully made this generalization were also more likely to have worse memory for details of the learning experience (i.e., the context), compared to those who did not make this generalization^22^. Yet, this trade-off between generalization and detailed memory has not been consistently reported^24,25^ and thus requires further investigation as to the parameters that drive this trade-off. In our study, we tested the following hypothesis: if the presence of conceptual knowledge promotes generalization during learning through episodic memory, it will impair the alternate function of episodic memory, to form detailed memories of that same information.

## Current study

The current study aimed to test two important questions about memory generalization. The first question is whether conceptual relatedness facilitates memory generalization, providing insight into how semantic knowledge drives this episodic memory function. The second question is whether generalization enhancements from conceptual relatedness come at the cost of forming detailed memory representations, another important function of episodic memory. To meet our study aim, we modified the acquired equivalence task and paired it with a recognition memory test. During this modified acquired equivalence task, participants were required to directly learn overlapping object-scenes pairs (A–X, B-X and A-Y). Among all overlapping objects (A-B), half belonged to the same conceptual category (conceptual condition, e.g. A: pencil—office supplies; B: scissors—office supplies) and the other half to different conceptual categories (neutral condition, e.g. A: watch—accessories; B: fork—kitchen items). We tested how participants would generalize the acquired knowledge to make inferences about indirectly learned pairs (B–Y) as well as to novel but conceptually related items, which was done by introducing new objects (C) that were semantically related to the first encountered objects (e.g., A—pencil, C—ruler). The recognition memory test measured the ability to identify objects (and scenes in one experiment) encountered on the acquired equivalence task and discriminate these items from similar lures, a strong test of detailed memory recollection^26^. Our first prediction was that if conceptual relatedness facilitates generalization, then participants would make more correct inferences to indirectly learned pairs from the conceptual condition. Our second prediction was that if there is a trade-off between specificity and enhanced generalization from conceptual trials, we expected this enhanced rate of generalization to be accompanied by poorer recognition memory for items from the conceptual than neutral condition.

## Experiment 1

### Experimental overview

All methods were approved by and carried out in accordance with relevant guidelines and regulations set forth by the McGill University Ethics Review Board and in accordance with the Declaration of Helsinki. Informed consent was obtained from all participants prior to beginning the experiment.

**Figure 1:**
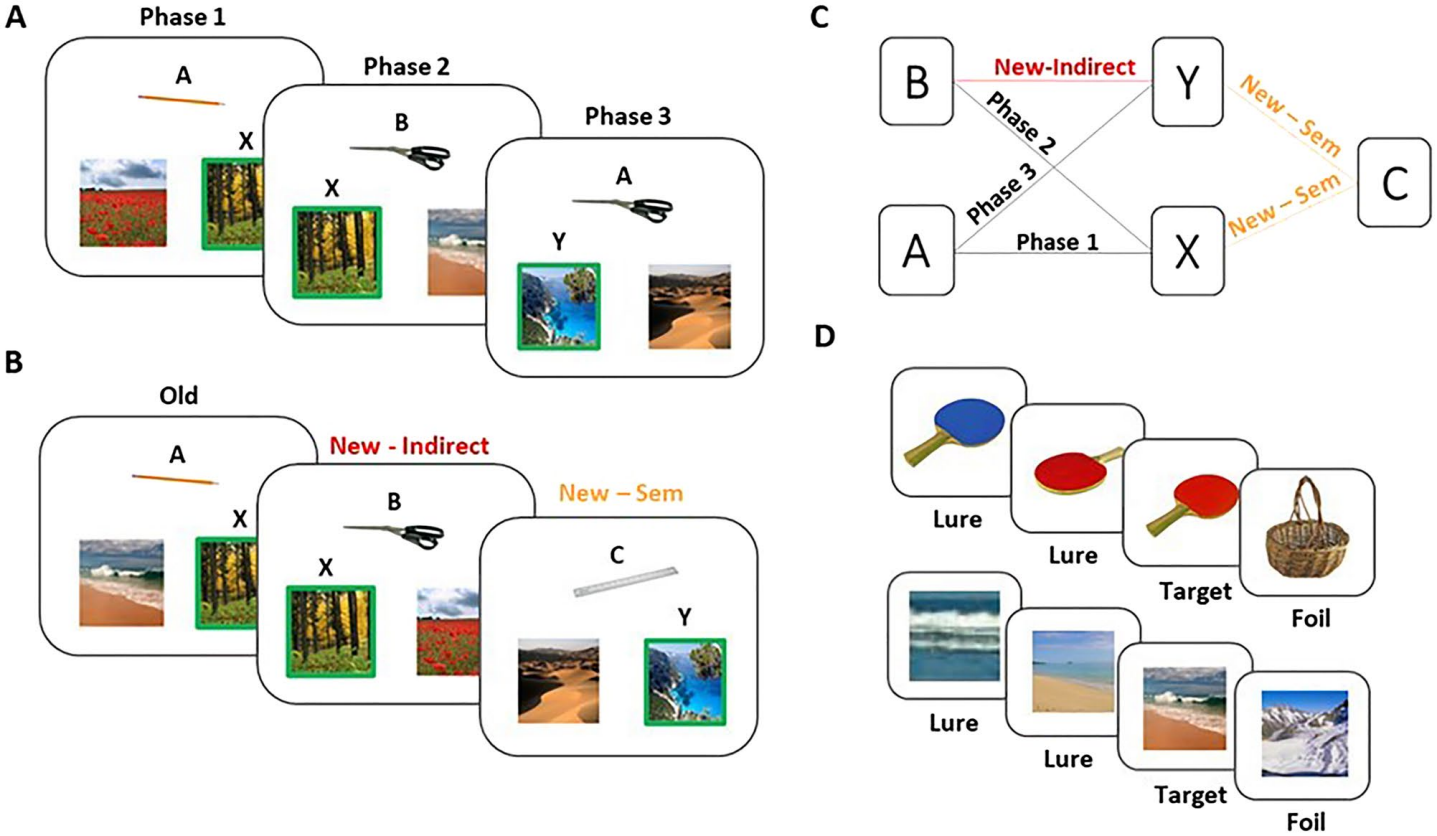
Structure of the acquired equivalence task and the recognition memory test. (**A**) Participants first learned object-scene pairs (A–X, B–X and A–Y) across three phases of learning. In the conceptual condition, half of the object-scene pairs included objects from the same concept category (e.g., A—pencil; B—scissors) while in the neutral condition, all object pairs were from different categories (e.g., A—keyboard; B—toothbrush). (**B**) During test, participants are required to infer new object-scene pairs that were indirectly learned (generalization trials B–Y) but also pairs of new C objects that are conceptually related to the object A, presented with the two scenes associated with A (C–X and C–Y). (**C**) Structure of the overlapping object-scene pairs, directly through the three phases of learning (Phase 1, A–X, Phase 2, B–X, and Phase 3, A–Y), indirectly learned pairs (New; B–Y), as well as new and semantically related pairs (New-Sem; C-X, C-Y). (**D**) Participants made old/new recognition decisions about each object (Targets) as well as others that were slight perceptual modifications of those that were learned (Lures) and completely new items (Foils).

Participants completed a modified version of the acquired equivalence task, consisting of three learning phases and a test phase for memory generalization, followed by a recognition memory test for items from the acquired equivalence task (see Fig. 1 for a schematic of the experimental design). In the acquired equivalence learning phases, participants encoded a series of object-scene pairs, either directly or indirectly. We manipulated the conceptual relatedness of these object pairings such that half of the object pairings were from the same concept category and the other half were from unrelated concept categories. During the acquired equivalence test phase, participants’ memory for the learned (old) object-scene pairs as well as their ability to infer the (new) indirectly learned object-scene pairs (generalization) were tested. In this phase, we also introduced new semantically related objects that were paired with the scenes. The recognition memory test that followed included the objects and scenes from the acquired equivalence task (old) as well as new objects and scenes that were lures (perceptually similar items to those of the acquired equivalence task) or completely novel items (foils).

### Methods

#### Participants

Twenty-four undergraduate student participants were recruited from McGill University’s Psychology Participant Pool or online advertisements (*M*_age_ = 21.04 years, range = 19 to 24 years, *SD*_*age*_ = 1.57; 19 females, 5 males). This sample size was based on prior research that used a similar experimental paradigm^27^. All participants were fluent English speakers, free from known neurological or psychological conditions, and had normal or corrected-to-normal vision and hearing. The study protocol was approved by the Ethics Review Board of McGill University. All participants gave informed consent before taking part in the study and received course credit for their participation or were monetarily compensated for their time.

#### Stimuli

Images of common objects and outdoor scenes were selected from stimuli sets developed by Konkle and colleagues^28,29^. For the acquired equivalence learning phases, 16 images of outdoor scenes were selected, as well as eight pairs of conceptually unrelated objects (i.e., objects from different superordinate categories; *keyboard* and *toothbrush*; s*crewdriver* and *leaf*; *apple* and *chair*; *fork* and *watch*) and eight pairs of conceptually related objects (i.e., from the same superordinate category; office supplies: *pencil* and *scissor*; clothing: *t-shirt* and *pants*; musical instruments: *trumpet* and *guitar*; sports items: *table tennis paddle* and *baseball*). Eight additional objects from the same conceptual category as the first item of the conceptually unrelated object pairs (electronics: *computer mouse* with *keyboard*; tools: *hammer* with *screwdriver*; fruit: *banana* with *apple*; kitchen items: *wooden spoon* with *fork*) and the conceptually related object pairs (office supplies: *ruler* with *pencil*; clothing: *shirt* with *t-shirt*; musical instruments: *violin* with *guitar*; sports items: *basketball ball* with *baseball ball*) were selected to serve as new semantic pairs in the acquired equivalence test phase.

For the object recognition memory test, we selected two additional objects that were perceptually similar to each object from the acquired equivalence task (n = 48) to serve as lure items, and 24 additional objects unrelated to the objects from the acquired equivalence task (n = 24) to serve as foils. For the scene recognition memory test, we selected 32 scenes that were similar to the images used in the acquired equivalence task (lure items) and 16 completely novel outdoor scenes (foil items).

#### Procedure

The experiment was presented using the Psychophysics Toolbox (http://psychtoolbox.org/)^30^ for MATLAB (MathWorks, Inc., Natick, MA). Over a single experimental session, participants completed three learning phases and the test phase of the acquired equivalence task followed by a recognition memory test.

#### Acquired equivalence task

In the first learning phase, participants learned pairs between eight objects (A) and eight scenes (X). On each trial, participants were presented with an image of an object (A) in the center of the screen along with two images of scenes below (X_1_ and X_2_). They were asked to select the scene they believed to be paired with the object via button response and received feedback for 1.2 s indicating the correct scene paired with the object. This was followed by a fixation-cross for 500 ms. Participants had no time limit to indicate their responses. During each learning and testing trial, the “incorrect scene” that was presented on the screen with the “correct scene” was randomly selected from the pool of scenes introduced during the specific learning phase (i.e., in the first learning, four scenes were introduced, one of the scenes is the “correct scene” while the “incorrect scene” wass randomly selected from the remaining three scenes).

In the second learning phase, participants were presented with a different set of object-scene pairs that followed the same trial structure as phase one. In this phase, they learned pairings between the previously eight encountered scenes (X) with eight new objects (B). Half of these B objects were conceptually related to the A object previously paired with scene X (conceptual condition; e.g., if A was a *pencil*, then B could be *scissors*, both items from the concept category ‘office supplies’) and half were unrelated to the associated A object (neutral condition; e.g., if A was a *keyboard*, then B would be a *toothbrush,* objects from different concept categories of ‘office supplies’ and ‘bathroom items’).

In the third learning phase, following the same trial structure as prior phases, participants learned new pairings between objects from the first phase (A) and eight new scenes (Y). For each learning phase, each object-scene pair was presented randomly six times and the previously learned pairs were randomly presented with the novel pairs in each learning phase.

The testing phase took place after a 10 min filled delay and assessed memory for directly learned object-scene pairs (A–X, B–X, A–Y) and the ability to infer object-scene pairings for new indirectly learned object-scenes (B–Y) and for object-scene pairs that included new objects (C–Y and C–X) that were not present in any of the learning phases but were conceptually related to the objects from the first learning phase (A objects; if A was a *keyboard*, then C could be a *computer mouse*). If the participants engaged in generalization, they should show a bias towards selecting the scene that was associated with the related object, established through indirect learning (B-Y through learning A–X, B–X, and A–Y) or prior knowledge (C–Y and C–X through learning A–X and A–Y). As done in prior work, each of the object-scene pairs (A–X, B–X, A–Y, B–Y, C–X, and C–Y) was presented six times over the session for a total of 288 trials^4^. The trial structure for this test phase was similar to the learning phases. Participants were presented with an object with two scenes below and asked to select the scene they thought would be paired with the object. Participants were told to base their decisions on what was learned earlier in the experiment and no feedback was given.

#### Recognition memory test

Directly after the acquired equivalence task, participants completed an object then a scene recognition memory test. In the object recognition test, a series of 64 object images was randomly presented on the computer screen and participants were asked to decide if each image of an object was old (from the acquired equivalence task) or new (not seen in the previous task), indicating their response via a button press with no time limit. Sixteen objects were those from the acquired equivalence task (objects A, B, C; ‘old’ targets), 32 objects were similar to each of those from the acquired equivalence task (two lures for each object A, B, C) and 16 objects were completely new objects (foils), following prior recognition memory test designs^31^. In the scene recognition memory test, participants made the same old/new decisions to images of the 16 scenes from the acquired equivalence task (‘old’ targets), 32 scenes similar to those used in the acquired equivalence task (e.g., different color or orientation; lures), and 16 images of new unrelated scenes (foils).

#### Statistical analysis

Analyses were conducted using the R statistical software (version 4.2.3; R Core Team, 2023^32^). For all our variables of interest, the absolute values of the Skewness and Kurtosis were within the acceptable range of < |3| and < |10| respectively^33^. Performance on the acquired equivalence task was measured as the proportion of correct responses given by participants, separately for learning and test phases. Our first analysis was a repeated measures ANOVA on test performance with condition (neutral, conceptual) and trial type (old, new) as factors. The old trial type refer to all the object-scene pairs previously learned, while the new trial type refer to the new indirectly learned and the new semantic trials. We conducted follow-up ANOVAs separately for the old (A–X, B–X, and A–Y) and new trial types ( (B–Y and C–X, C–Y)). Finally, we ran a repeated measures ANOVA to assess if learning performance was different across condition (conceptual, neutral) as a function of the learning phase (first, second, and third). Here, we focused the analysis on the novel pairs introduced at each learning phase (i.e., A–X during phase one, B–X during phase two, A–Y during phase three). Bonferroni corrected post-hoc tests were run to follow up on any significant main or interaction effects from these ANOVAs.

For the recognition memory test, repeated measures ANOVAs with condition (conceptual, neutral) and object type (A, B, C) were run on proportion of correct responses to *target* items, the percentage of “old” responses given to targets, which we refer to as hits, as well the number of “old” responses given to *lures*, which we refer to as false alarms. These are both important metrics of detailed memory, with hits indicating memory accuracy and false alarms indicating the precision of memory^34^. Bonferroni corrected post-hoc tests were run to follow up on any significant main or interaction effects. For the scene recognition memory test, we have conducted two pairwise t-tests comparing hits and false alarms between condition.

### Results

#### Acquired equivalence task

Table 1 reports the summary statistics during the learning and testing phases by condition (conceptual, neutral). Focusing on the test phase, we first ran a repeated measures ANOVA on test performance as a function of trial (old, new) and condition (conceptual, neutral), which revealed a main effect of trial (*F*(1, 23) = 28.38, *p* < 0.001, partial η^2^ = 0.55), a marginally significant main effect of condition (*F*(1,23) = 4.37, *p* = 0.05, partial η^2^ = 0.16), and a significant trial x condition interaction (*F*(1,23) = 6.95, *p* = 0.01, partial η^2^ = 0.23). Bonferroni corrected post-hoc analysis revealed no differences between conceptual and neutral conditions for the old pairs (*t*(23) = − 0.98, *p* = 0.67, *d* = − 0.2, 95% CI [− 0.01, 0.01]), however, a higher performance—more generalization—for the conceptual (*M* = 0.88, *SD* = 0.11) than neutral condition (*M* = 0.85, *SD* = 0.11; t(23) = 2.5, *p* = 0.02, *d* = 0.51, 95% CI [0.01, 0.06]) was found.

**Table 1:** Mean accuracy performance during the acquired equivalence task in Experiment 1.

Performance	Conceptual	Neutral
Learning		
Phase 1—A–X	0.81 (0.15)	0.88 (0.09)
Phase 2—B–X	0.85 (0.13)	0.88 (0.07)
Phase 3—A–Y	0.82 (0.12)	0.85 (0.09)
Testing		
Old—A–X	0.95 (0.04)	0.96 (0.07)
Old—B–X	0.95 (0.05)	0.95 (0.05)
Old—A–Y	0.92 (0.09)	0.92 (0.09)
New—indirect B–Y	0.86 (0.13)	0.75 (0.15)
New—semantic C–X	0.91 (0.11)	0.91 (0.11)
New—semantic C–Y	0.88 (0.13)	0.89 (0.13)

Values in parentheses are standard deviations. The performance for learning reflects those for the novel pairs introduced during that phase of learning. The performance for testing reflects old, directly learned pairs encountered in the previous phases of learning as well as new pairs, those indirectly learned through learning (B–Y) and those that include semantically-related C objects.

Since the new trials included both indirectly learned pairs (B–Y) and semantically related pairs (C–X and C–Y), we ran a follow-up ANOVA on generalization (test) performance for new object-scenes pairs as a function of pair type (indirect B–Y, semantic pair C–X, semantic pair C–Y) and condition (conceptual, neutral), which revealed a main effect of pair type (*F*(2, 46) = 27.38, *p* < 0.001, partial η^2^ = 0.54), a significant main effect of condition (*F*(1, 23) = 6.26, *p* = 0.02, partial η^2^ = 0.21), and a significant pair type x condition interaction (*F*(12, 46) = 7.92, *p* = 0.001, partial η^2^ = 0.26). Bonferroni corrected post-hoc analysis revealed higher accuracy for indirect (B–Y) pairs in the conceptual than neutral condition (*t*(23) = 4.08, *p* < 0.001, *d* = 0.83, 95% CI [0.05, 0.17]), while no difference was found between conceptual and neutral trials for the semantic (C–X and C–Y) pairs (all *p*-values > 0.60; see Fig. 3).

The repeated-measure ANOVA on learning performance as a function of condition and learning phase (first, second, and third) revealed a main effect of condition (*F*(2,46) = 6.99, *p* = 0.01, partial η^2^ = 0.23) with better performance while learning in the neutral (*M* = 0.87, *SD* = 0.08) than conceptual condition (*M* = 0.83, *SD* = 0.13), but no significant main effect of the learning phase, nor interactions between these factors (all *p-*values > 0.39).

#### Object recognition memory test

**Figure 2:**
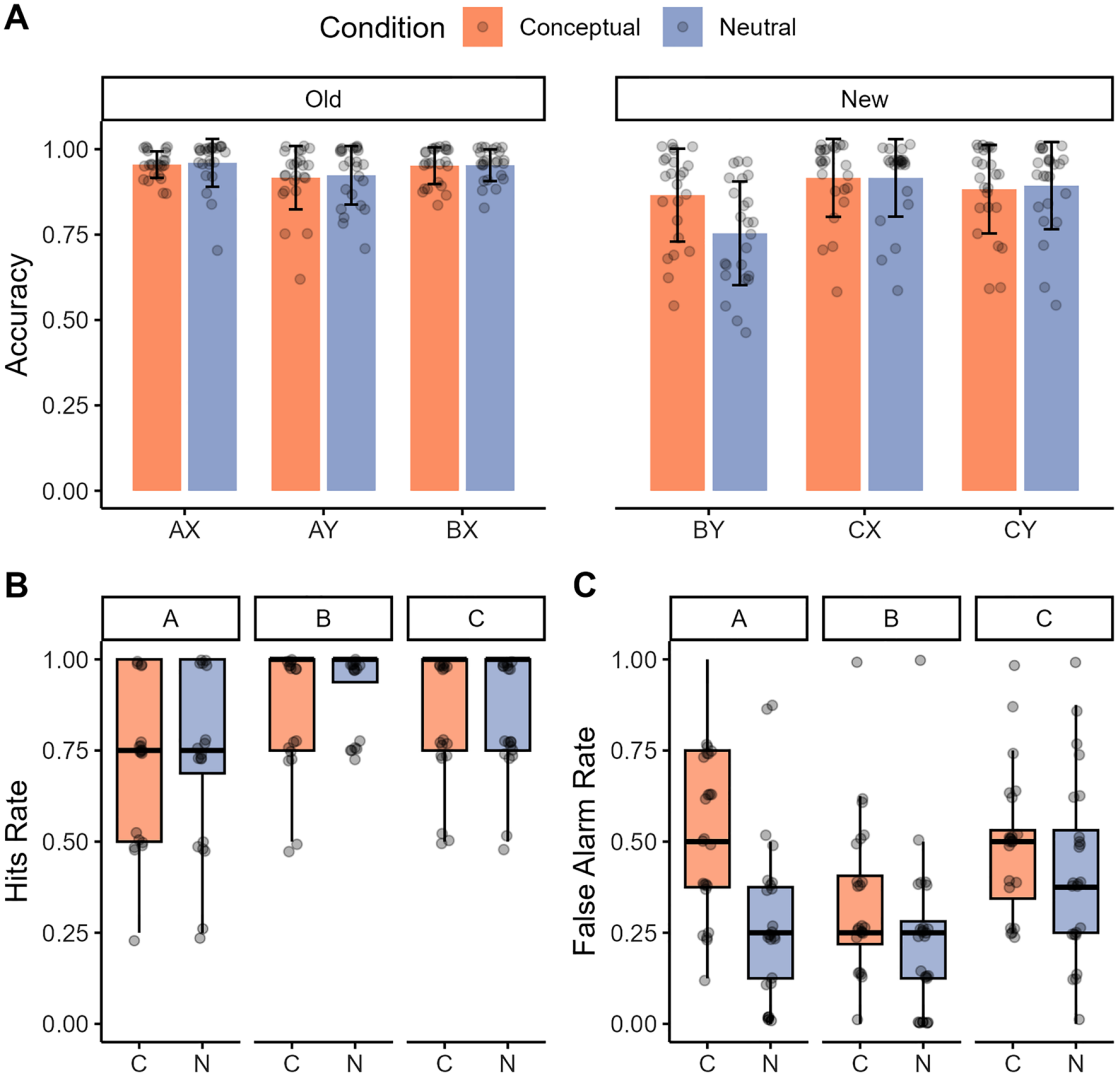
Impact of condition on memory generalization (**A**) and object recognition memory (**B**) in Experiment 1. (**A**) Bar plots of the accuracy (proportion correct response) for old (pairs acquired during learning: A–X, B–X, B–Y), new (generalization trials that are indirectly learned: B–Y; trials that are semantically related to the initially learned objects and paired with associated scenes, C–X, C–Y) during the test phase of the acquired equivalence task. Error bars denote standard error of the mean. (**B**) Boxplot of the hit rates for encountered (old) objects A, B and C on the recognition memory test. The error bars denote standard error of the mean and points are individual data points. (**C**) Boxplots of the rate of false alarm for lure objects A, B and C on the recognition memory test. The error bars denote standard error of the mean and points are individual data points.

The responses given by participants for the target, foil and lure objects indicate that participants were generally able to correctly identify the target as old (hits; *M* = 0.85, *SD* = 0.13), the foils as new (*M* = 0.99, *SD* = 0.02), but had more difficulty identifying the lure objects, indicated by the high false alarm rates (proportion of old response) to the lure objects (*M* = 0.62, *SD* = 0.19). To understand this pattern, we first ran an ANOVA on the average hit rates as a function of object type (A, B and C objects introduced during first learning, second learning or at test, respectively) and condition (conceptual, neutral). This ANOVA revealed a main effect of object type (*F*(2,46) = 8.17, *p* = 0.001, partial η^2^ = 0.26), with higher hit rates for object B (*M* = 0.92, *SD* = 0.14) than object A (*M* = 0.77, *SD* = 0.23; *t*(47) = 3.93, *p* < 0.001, *d* = 0.63, 95% CI [− 0.22, − 0.07]), but no significant main effect of condition nor a significant interaction between object type and condition (all *p-*values > 0.46). A similar ANOVA on false alarm rates, however, revealed a main effect of object type (*F*(2, 64) = 23.45, *p* < 0.001; partial η^2^ = 0.50), a main effect of condition (*F*(1, 23) = 36.33, *p* < 0.001; partial η^2^ = 0.61), and an interaction between these factors (*F*(2, 64) = 5.95, *p* = 0.005; partial η^2^ = 0.21). Participants had lower rates of false alarms for the lures related to object B in the neutral (*M* = 0.32, *SD* = 0.22) than conceptual condition (*M* = 0.23, SD = 0.22; *t*(23) = − 2.99, *p* = 0.031, *d* = 0.61, 95% CI [0.03, 0.15]). Participants also had lower rates of false alarms for the lures related to object A in the neutral (*M* = 0.29, *SD* = 0.23) than conceptual condition (*M* = 0.52, *SD* = 0.22; *t*(23) = 5.69, *p* < 0.001, *d* = 1.16, 95% CI [0.15, 0.31]), but no condition difference was found for the lures related to objects C (*p* = 0.22; see Fig. 2).

#### Scene recognition memory test

The responses given by participants for the target, foil and lure scenes indicate that participants were able to identify target scenes as old (*M* = 0.93, *SD* = 0.10), scene foils as new (*M* = 0.99, *SD* = 0.02) and scene lures as new (*M* = 0.93, *SD* = 0.09). The t-tests for hit and false alarm rates as a function of condition (conceptual vs. neutral) revealed no significant effect of condition (all *p*-values > 0.69).

### Experiment 1: summary

Consistent with our first prediction, we found evidence that memory generalization, the likelihood to infer a relationship between experienced events, is enhanced when there is shared conceptual knowledge amongst those events. Indeed, when objects A and B were conceptually related (conceptual condition), and participants learned that object A was paired with scene Y, participants were better at inferring the association between object B and scene Y than when the objects were not conceptually related (neutral condition). We also found that semantic or conceptual knowledge enhanced generalization beyond learned information. When participants encountered novel objects C- those that were semantically related to the learned objects A - during the test phase of the acquired equivalence task, they were likely to infer an association with the scenes paired with the semantically related object they did encounter during learning. Also, consistent with our second prediction, we found that the boost in generalization from shared conceptual knowledge came at the expense of detailed memory for those objects, as there were higher rates of false alarms to lures related to objects from the conceptual condition compared to those from the neutral condition. To confirm and replicate these results, we conducted a second experiment. This second experiment was conducted online with timing modifications (described below). We also opted to remove the scene recognition memory test in this Experiment given the ceiling level performance found in Experiment 1.

## Experiment 2

### Methods

#### Participants

A prior calculation of the needed sample size to obtain a power of 0.8 that was based on the results from Experiment 1 revealed that a sample of 40 was needed. Due to the online nature of this experiment, we increased the sample by 15% to address potential dropouts and performance issues. A total of 47 young participants, between 18 and 36 years old, were recruited from a local cohort with the same inclusion criteria as Experiment 1. Participants were monetarily compensated for their time. Six participants were excluded due to below chance level performance at test, computed using binomial distribution set at 0.57. Thus, the final analyzed sample consisted of 41 participants (34 female, 6 male, and 1 non-binary; *M*_age_ = 21.63, *SD*_age_ = 3.16, *M*_edu_ = 14.68, *SD*_edu_ = 1.75).

#### Materials and procedure

The same acquired equivalence task and object recognition memory test as Experiment 1 were used but programmed using Gorilla Experiment Builder^35^ for online data collection. One notable difference between Experiments 1 and 2 was the absence of the 10 min delay between the learning and testing phase of the acquired equivalence task, which was done to minimize uncontrolled behaviours during a delay, as this was an online experiment. The same analyses conducted for Experiment 1 were conducted for Experiment 2. Like Experiment 1, all methods were approved by the McGill University Ethics Review Board and in accordance with the Declaration of Helsinki and informed consent was obtained from all participants.

### Results

#### Acquired equivalence task

Table 2 reports the summary statistics during learning and testing phases by condition (conceptual, neutral). Similar to Experiment 1, we ran a repeated measures ANOVA on test performance as a function of trial type (old, new) and condition (conceptual, neutral), which revealed a main effect of trial type (*F*(1,40) = 86.44, *p* < 0.001, partial η^2^ = 0.68), a significant main effect of condition (*F*(1,40) = 48.07, *p* < 0.001, partial η^2^ = 0.55), and a significant trial type × condition interaction (*F*(1,40) = 38.99, *p* < 0.001, partial η^2^ = 0.49). Bonferroni corrected post-hoc analysis revealed no differences between conditions on the old trials (*p* = 0.06) but higher accuracy in the conceptual (*M* = 0.87, *SD* = 0.12) than the neutral condition for the new trials (*M* = 0.74, *SD* = 0.16; *t*(40) = 7.03, *p* < 0.001, *d* = 1.10, 95% CI [0.09, 0.17]). To follow up on these results, we ran a repeated measures ANOVA on average response accuracy for new pairs as a function of condition and pair type (indirect B–Y, semantic C–X and semantic C–Y), which revealed a main effect of condition (*F*(1,40) = 49.41, *p* < 0.001, partial η2 = 0.55), with higher performance for conceptual (*M* = 0.87, *SD* = 0.15) than neutral condition (*M* = 0.74, *SD* = 0.18), a main effect of pair type (*F*(1,40) = 421.22, *p* < 0.001, partial η^2^ = 0.35), with higher accuracy for semantic C–X pairs (*M* = 0.86, *SD* = 0.15) when compared to indirect B–Y pairs (*M* = 0.77, *SD* = 0.19; *t*(40) = − 5.86, *p* < 0.001, *d* = − 0.65, 95% CI [− 0.12, − 0.06]) and to semantic C–Y pairs (*M* = 0.78, *SD* = 0.18; *t*(40) = 5.23, *p* < 0.001, *d* = 0.58, 95% CI [0.05, 0.11]), but no interaction between these factors (*p* = 0.38).

**Table 2:** Accuracy (proportion correct response) during the acquired equivalence task in Experiment 2.

Performance	Conceptual	Neutral
Learning		
Phase 1—A–X	0.83 (0.14)	0.81 (0.13)
Phase 2—B–X	0.87 (0.10)	0.78 (0.14)
Phase 3—A–Y	0.84 (0.10)	0.82 (0.11)
Testing		
Old—A–X	0.97 (0.04)	0.96 (0.06)
Old—B–X	0.96 (0.05)	0.94 (0.07)
Old—A–Y	0.92 (0.10)	0.91 (0.09)
New—indirect B–Y	0.85 (0.15)	0.70 (0.20)
New—semantic C–X	0.92 (0.13)	0.81 (0.15)
New—semantic C–Y	0.85 (0.14)	0.71 (0.19)

Values in parentheses are standard deviations. The performance for learning reflects those for the novel pairs introduced during that phase of learning. The performance for testing reflects old, directly learned pairs encountered in the previous phases of learning as well as new pairs, those indirectly learned through learning (B–Y) and those that include semantically-related C objects.

Finally, the repeated-measure ANOVA on learning performance as a function of condition and learning phase revealed a main effect of condition (*F*(2,80) = 18.07, *p* < 0.001, partial η^2^ = 0.31) and an interaction between condition and learning phase (*F*(2,80) = 4.73, *p* = 0.01, partial η^2^ = 0.11), but no main effect of learning phase (*p* = 0.61). Participants showed better learning for the conceptual (*M* = 0.87, *SD* = 0.10) than neutral condition (*M* = 0.78, *SD* = 0.14; *t*(40) = 4.34, *p* < 0.001, *d* = 0.68, 95% CI [0.05, 0.12]) in the second phase of learning, but not in the other phases (all *p*-values > 0.08).

#### Object recognition memory test

**Figure 3:**
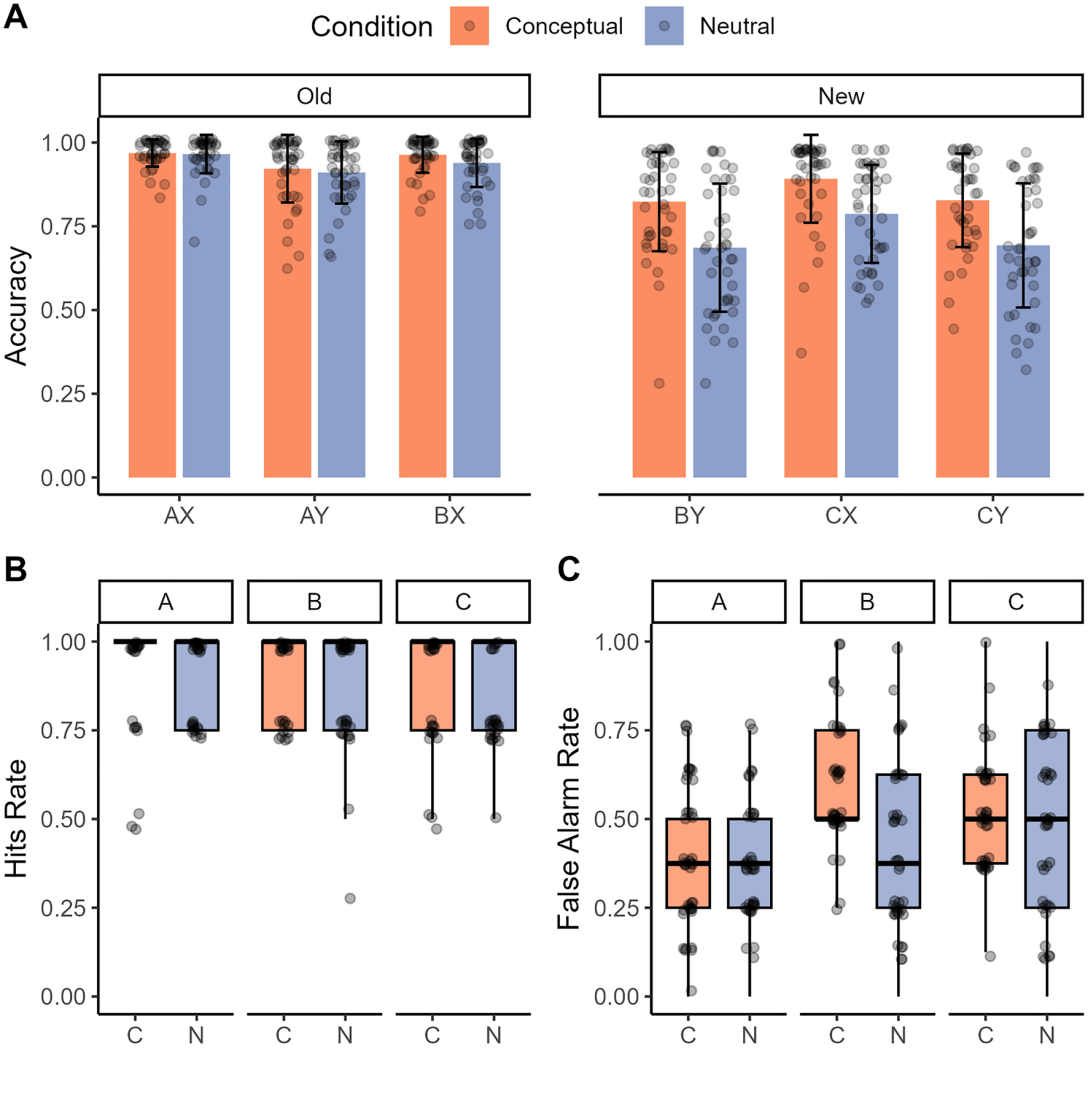
Experiment 2. (**A**) Bar plots of the accuracy (proportion correct response) for old (pairs acquired during learning: A–X, B–X, B–Y), new (generalization trials that are indirectly learned: B–Y; trials that are semantically related to the initially learned objects and paired with associated scenes, C–X, C–Y) during the test phase of the acquired equivalence task. Error bars denote the standard error of the mean. (**B**) Boxplots of the hit rates for encountered (old) objects A, B and C on the recognition memory test. The error bars denote standard error of the mean and points are individual data points. (**C**) Boxplots of the rate of false alarms for lure objects A, B and C on the recognition memory test. The error bars denote standard error of the mean and points are individual data points.

The responses given by participants for the target, foil and lure objects indicate that participants are generally able to correctly identify the targets as old (*M* = 0.91, *SD* = 0.09), the foils as new (*M* = 0.99, *SD* = 0.03), but had more difficulty identifying the lure objects as new, as indicated by the high false alarm rates (old response) to the lure objects (*M* = 0.44, *SD* = 0.18). The ANOVA on average hit rates as a function of condition (conceptual, neutral) and object type (A, B, C) did not reveal significant main effects of condition or object type nor interaction between these factors (all *p-*values > 0.09). The same ANOVA on false alarms rates, however, revealed a main effect of condition (*F*(1,40) = 15.55, *p* < 0.001, partial η^2^ = 0.28), a main effect of object type (*F*(2,80) = 33.53, *p* < 0.001, partial η^2^ = 0.46), and a significant interaction between condition and object type (*F*(2,80) = 12.47, *p* < 0.001, partial η^2^ = 0.24). Participants showed higher false alarm rates for the B objects (*M* = 0.51, *SD* = 0.21; *t*(81) = − 6.36, *p* < 0.001, *d* = 0.70, 95% CI [− 0.18, − 0.09]) and the C objects (*M* = 0.52, *SD* = 0.23; *t*(65) = − 6.96, *p* < 0.001, *d* = 0.77, 95% CI [− 0.18, − 0.10]) when compared to the A objects (*M* = 0.37, *SD* = 0.22). Importantly, participants showed higher false alarm rates for the B objects from the conceptual (*M* = 0.61, *SD* = 0.18) compared to the neutral condition (*M* = 0.42, *SD* = 0.24; *t*(40) = 6.59, *p* < 0.001, *d* = 1.03, 95% CI [0.13, 0.25]; see Fig. 3). There was no difference between conditions for the A or the C objects (*p*-values > 0.95).

### Experiment 2: summary

As in Experiment 1, we found enhanced generalization yet reduced recognition memory (indexed by increased false alarm rates) for conceptually related objects in the acquired equivalence task. In this experiment, we found enhanced generalization of information that was presented multiple times in the acquired equivalence task (A–X, B–X generalized to C–X) but not for information learned later on during the task, and only for one object (A–Y did not generalize as well to B–Y and C–Y). Additionally, while in Experiment 1 the lures associated with the B objects, those introduced later in the learning phases, had the lowest false alarm rates, here, we found that these objects were associated with higher false alarm rates than objects A, those introduced in the earliest learning phase of the acquired equivalence task. Thus, while Experiment 2 replicated the main findings from Experiment 1 and further confirmed our predictions, these unexpected differences warranted further investigation. Of note, our prior two experiments had some important methodological differences, such as the testing environment (lab vs. online) and the delay between learning and testing in the acquired equivalence task (10 min vs. no delay). Thus, we conducted a third online Experiment replicating Experiment 2 but with the same delay between learning and testing as in Experiment 1.

## Experiment 3

### Methods

#### Participants

Using the same rationale for sample size and recruitment method as Experiment 2, 45 young participants, between 18 and 36 years old, were recruited from a local cohort. From the total sample, 12 participants were excluded due to below chance level learning and recall of learned pairs. Thus, the final analyzed sample consisted of 33 participants (30 female, 2 male, 1 prefer not to say; *M*_age_ = 20.42, *SD*_age_ = 1.30, *M*_edu_ = 13.69, *SD*_edu_ = 1.53).

#### Materials and procedure

The structure of the acquired equivalence task followed Experiment 2 but with a 5 min filled delay between the learning and test phase in which participants filled out a demographic questionnaire and the Survey of Autobiographical Memory (SAM)^36^. The recognition memory test followed the same design as previous experiments. The same analyses conducted for Experiments 2 and 1 were conducted for Experiment 3. All methods were approved by the McGill University Ethics Review Board and in accordance with the Declaration of Helsinki and informed consent was obtained from all participants.

### Results

#### Acquired equivalence task

Table 3 shows an overview of summary statistics during learning and testing phases by condition. The first repeated measures ANOVA on test performance revealed a main effect of trial type (old, new; *F*(1,32) = 87.13, *p* < 0.001, partial η^2^ = 0.73), a significant main effect of condition (conceptual, neutral; *F*(1,32) = 59.83, *p* < 0.001, partial η^2^ = 0.65), and a significant trial type x condition interaction (*F*(1,32) = 55.48, *p* < 0.001, partial η^2^ = 0.63). Bonferroni corrected post-hoc analysis revealed no differences between conceptual and neutral conditions for the old trials (*p* = 0.06), however, higher accuracy was found for new trials in the conceptual (*M* = 0.91, *SD* = 0.10) than neutral condition (*M* = 0.77, *SD* = 0.12; *t*(32) = 8.03, *p* < 0.001, *d* = 1.40, 95% CI [0.10, 0.18]). The follow-up ANOVA on generalization performance for the new trials revealed a main effect of condition (*F*(1,32) = 64.46, *p* < 0.001, partial η^2^ = 0.67), with higher accuracy for conceptual (*M* = 0.91, *SD* = 11) than neutral condition (*M* = 0.77, *SD* = 0.15), a main effect of pair type (*F*(2,64) = 19.23, *p* < 0.001, partial η^2^ = 0.37), with higher accuracy for semantic C–X pairs (*M* = 0.89, *SD* = 0.12) than indirect B–Y pairs (*M* = 0.82, *SD* = 0.15; *t*(65) = − 5.08, *p* < 0.001, *d* = − 0.62, 95% CI [− 0.10, − 0.05]) and semantic C-Y pairs (M = 0.81, SD = 0.17; *t*(65) = 4.91, *p* < 0.001, *d* = 0.60, 95% CI [0.05, 0.12]), but no significant interaction between these factors (*p* > 0.05).

**Table 3:** Accuracy during the acquired equivalence task in Experiment 3.

Performance	Conceptual	Neutral
Learning		
Phase 1—A–X	0.85 (0.08)	0.88 (0.10)
Phase 2—B–X	0.88 (0.08)	0.82 (0.11)
Phase 3—A–Y	0.84 (0.12)	0.80 (0.11)
Testing		
Old—A–X	0.97 (0.04)	0.96 (0.06)
Old—B–X	0.96 (0.05)	0.94 (0.07)
Old—A–Y	0.92 (0.10)	0.91 (0.09)
New—indirect B–Y	0.85 (0.15)	0.70 (0.20)
New—semantic C–X	0.92 (0.13)	0.81 (0.15)
New—semantic C–Y	0.85 (0.14)	0.71 (0.19)

Values in parentheses are standard deviations. The performance for learning reflects those for the novel pairs introduced during that phase of learning. The performance for testing reflects old, directly learned pairs encountered in the previous phases of learning as well as new pairs, those indirectly learned through learning (B-Y) and those that include semantically-related C objects.

The repeated-measure ANOVA on learning performance revealed a main effect of condition (*F*(1,32) = 10.82, *p* = 0.002, partial η^2^ = 0.25), a main effect of learning phase (first, second, third; *F*(2,64) = 6.19, *p* = 0.003, partial η^2^ = 0.16), and a significant interaction between learning phase and condition (*F*(2,64) = 8.49, *p* = 0.001, partial η^2^ = 0.21. The interaction effect was due to better learning in the conceptual condition in the second phase of learning (*M* = 0.90, *SD* = 0.07) when compared to the neutral condition (*M* = 0.83, *SD* = 0.10; *t*(32) = 4.77, *p* < 0.001, *d* = 0.83, 95% CI [0.04, 0.10]), while no difference was found between conditions in the first and third phases of learning (all *p*-values > 0.09).

#### Object recognition memory test

The responses given by participants for the target, foil and lure objects indicate that participants are generally able to correctly identify the targets as old (*M* = 0.91, *SD* = 0.08), the foils as new (*M* = 0.99, *SD* = 0.01), but had more difficulty identifying the lure objects, indicated by the high false alarm rates (old response) to the lure objects (*M* = 0.50, *SD* = 0.18). The ANOVA on the average hit rates revealed a significant main effect of object type (A, B, C; *F*(2,64) = 4.26, *p* = 0.02, partial η^2^ = 0.12), with higher hit rates for A objects (M = 0.95, SD = 0.11) compared to B objects (*M* = 0.87, *SD* = 0.13, t(65) = 3.15, *p* = 0.002, *d* = 0.39, 95% CI [0.02, 0.10]), but no main effect of condition (conceptual, neutral) nor interaction between these factors (all *p-values* > 0.35).

**Figure 4:**
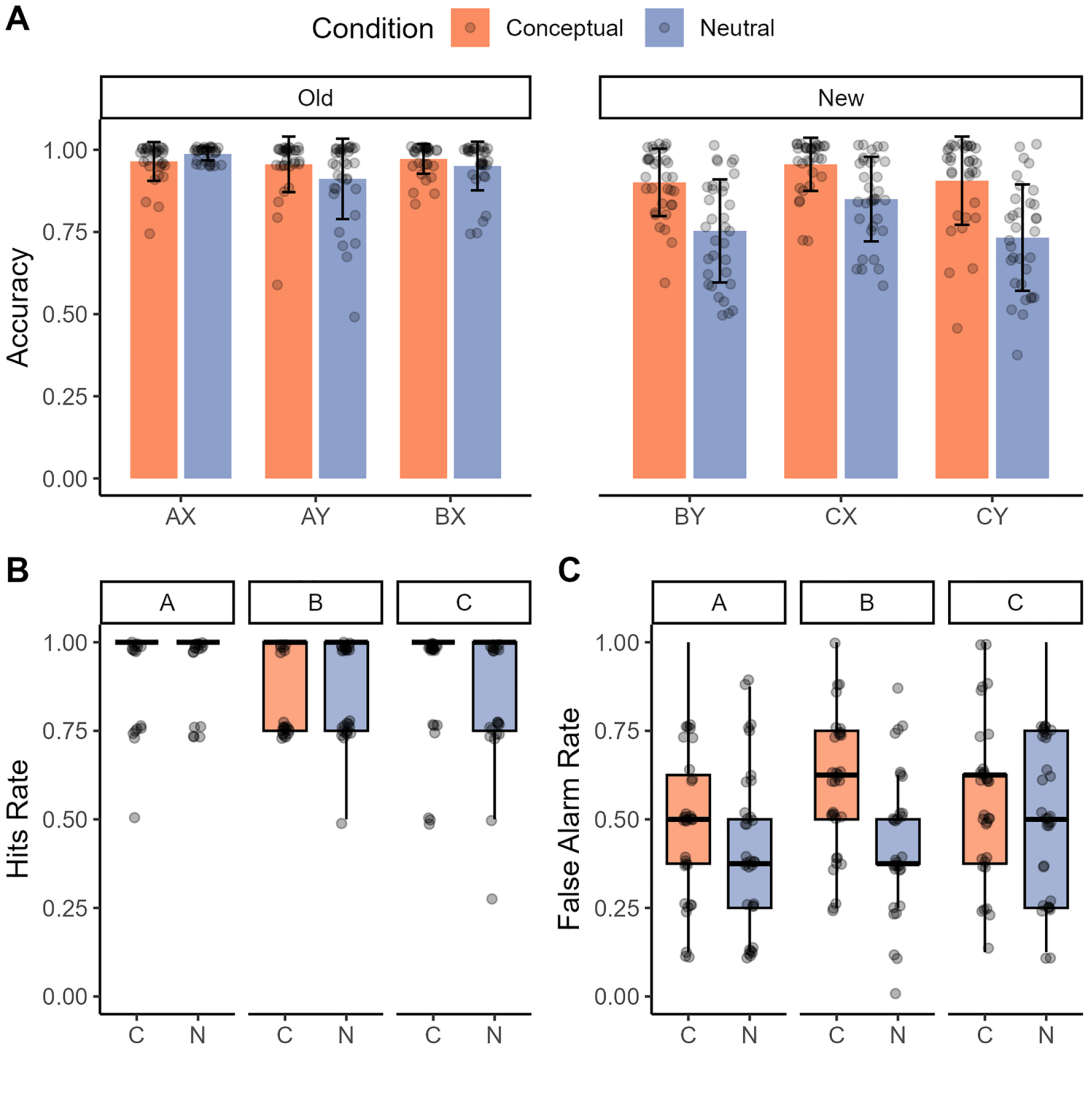
Impact of condition on memory generalization and item memory in Experiment 3. (**A**) Bar plots of the accuracy (proportion correct response) for old (pairs acquired during learning: A–X, B–X, B–Y), new (generalization trials that are indirectly learned: B–Y; trials that are conceptually related to the initially learned objects and paired with associated scenes, C–X, C–Y) during the test phase of the acquired equivalence task. Error bars denote the standard error of the mean. (**B**) Boxplots of the hit rates for encountered (old) objects A, B and C on the recognition memory test. The error bars denote standard error of the mean and points are individual data points. (**C**) Boxplots of the rate of false alarms for lure objects A, B and C on the recognition memory test. The error bars denote standard error of the mean and points are individual data points.

Replicating the prior Experiments, the same ANOVA on false alarm rates revealed a main effect of condition (*F*(1,32) = 25.76, *p* < 0.001, partial η^2^ = 0.45), a significant main effect of object type (*F*(2,64) = 8.55, *p* < 0.001, partial η^2^ = 0.21), but no interaction (*p* = 0.06). Participants had higher false alarms rates to lure objects from the conceptual (*M* = 0.54, *SD* = 0.21) than neutral condition (*M* = 0.46, *SD* = 0.23; see Fig. 4). Participants also had higher false alarm rates for the lures of the B objects (*M* = 0.52, *SD* = 0.22; *t*(65) = − 3.09, *p* = 0.003, *d* = 0.38, 95% CI [− 0.11, − 0.02]) and lures of the C objects (*M* = 0.54, *SD* = 0.22; *t*(65) = − 3.74, *p* < 0.001, *d* = 0.46, 95% CI [− 0.14, − 0.04]) when compared to the lures of the A objects (*M* = 0.45, *SD* = 0.22).

### Experiment 3: summary

Confirming our predictions and replicating the main results from the prior experiments, we found memory generalization was enhanced for objects that were conceptually related, for both indirectly learned pairs (replicating Experiment 1 and 2) and new semantically related items (replicating Experiment 2). We also found this benefit of generalization from conceptual relatedness presented alongside a reduced recognition memory performance, illustrated by a higher rate of false alarm for lures similar to objects that appeared in the conceptual than in the neutral condition. This pattern of results illustrates that generalization is associated with a less precise memory. Similar to Experiment 2, we also found lower rates of false alarms for lures linked to initially learned objects (A objects), which is different that the results from Experiment 1 in which we found lower rates of false alarms for lures linked to later learned objects (B objects). The possible reasons for this discrepancy are elaborated in the next section. Finally, regarding the methodological difference across Experiments, the similar pattern of results between Experiments 2 and 3 showed how the delay between learning and testing in the acquired equivalence task (delay vs. no delay) seems less relevant than the testing environment (lab vs. online) in driving performance.

### Conference presentation

Portions of these findings were presented as a poster at the 2021 Cognitive Neuroscience Society (CNS) Virtual Meeting.

## General discussion

The ability to generalize knowledge across shared experiences is a critical function of episodic memory, as is the ability to recall details from single experiences. Here, we aimed to answer two questions about the process of memory generalization. First, we sought to understand whether the ability to generalize knowledge across learned experiences is facilitated by the presence of shared conceptual knowledge. This question follows a long line of work acknowledging the impact of semantic (conceptual) processing on episodic memory (see^10^ for a review). Second, we sought to determine if promoting generalization through the presence of conceptual knowledge would be associated with a reduction in detailed memory, another fundamental function of episodic memory. Across three experiments, we modified a validated test of memory generalization, the acquired equivalence task, including a manipulation of conceptual relatedness. In this modified version, participants learned overlapping object-scene pairs that were conceptually related or unrelated to one another and we compared how participants generalized knowledge across these pair types as well as to new object-scene pairs that were only related semantically/conceptually (i.e., they were not learned during the task). We also gave participants a recognition memory test for the objects presented during the acquired equivalence task that included perceptually similar ‘lure’ objects, allowing us to determine if generalization ability was linked to a reduction in precise detailed memory (i.e., higher false alarms to these lure objects). Across all experiments, we found evidence that conceptually related information facilitated generalization across learned pairs as well as to new pairs, but was associated with a reduction in detailed memory, shown by higher false alarm rates to objects that shared conceptual information with others from the acquired equivalence task. We discuss the implications of these findings in the sections below.

### Conceptual information facilitates memory generalization

Our first main finding was that conceptual information led to higher rates of generalization on the acquired equivalence task. Broadly, this finding supports the notion that semantic processes, which underlie conceptual information, affect episodic memory functions^10–15^. This notion is also supported by prior work indicating that learning semantically related elements leads to better memory integration and generalization^18,19^.

More specifically, our results raise questions about how generalization judgements during acquired equivalence occur. One thought is that acquired equivalence results from individuals forming relationships among elements across experiences, and then using these relationships to form a generalized associated knowledge structure. From this structure generalizations and inferences about new experiences are made^4,6–9^. If this is the case, our finding that conceptual relatedness among elements common across experiences strengthened generalization suggests that conceptual knowledge is potentially strengthening the relationships amongst stored within this general structure. In other words, we think that conceptual information is stored within this general knowledge structure, thus promoting one to make inferences across conceptually-connected experiences and more broadly reflecting the interplay between semantic and episodic information^37,38^.

However, another explanation of our results is that the use of conceptual relatedness to inform generalization reflects the use of certain strategies  to make inferences. That is, participants could be simply using conceptual knowledge to strategically infer commonalities among separately stored memories rather than extracting information from an underlying general knowledge structure^39,40^. Future studies could test the nature of the processes driving the facilitation of generalization by conceptual knowledge, particularly whether these processes relate to  integrative encoding of general knowledge structures^4,6,7^ or strategic inferences implemented at test^39,40^. 

Our experiments also showed that the benefit of conceptual relatedness to generalization extended beyond learning. We found that participants were also more likely to use previously learned information (object-scene pairs) to make new inferences about object-scene pairs that were not learned, but that shared semantic content (i.e., conceptual features) with object of the learned object-scene pairs. Going back to the two mentioned possible explanations of generalization benefits from conceptual information, this finding is more likely to reflect a form of conceptual generalization based on strategic inference at the point of inference, as the presentation of these overlapping conceptual features was not introduced at encoding but only at test.  

Finally, some differences emerged across experiments with respect to the results on the acquired equivalence task. In the online Experiments 2 and 3, we found enhanced generalization abilities for novel yet semantically related objects in conceptual than neutral condition, but this effect was not evident in Experiment 1. This discrepancy could be linked to differences in testing (online versus in person) or the differences in learning across Experiments. While participants showed better learning of conceptually related pairs in Experiments 2 and 3, this was not observed in Experiment 1. Thus, one possibility is that when prior knowledge supports learning, presumably by strengthening the formation of integrated memory representations, this further supports generalization to new conceptually related information.

### Memory generalization at the expense of detailed memory

Across all experiments, we found evidence for a trade-off between engaging in memory generalization and memory specificity when conceptual knowledge was present. On the object recognition memory test that included target and lure objects for all object types presented in the acquired equivalence task, we found more false alarms (responding ‘old’ to lure objects) for objects from the conceptual condition. This finding aligns with previous work showing reduced detailed memory, manifested as more false memories and source attribution errors, for information encoded as part of general integrated memory representation^21,22^.

Our finding of a trade-off between generalization and specificity also indicates that these mnemonic functions might rely on orthogonal memory processes. Based on prior work, memory specificity might be related to pattern separation processes, in which similar events are stored in non-overlapping memory representations. In contrast, generalization might rely more on pattern completion processes, in which previously stored knowledge supports the formation of new memory representations^41^. When individuals encounter information associated with prior conceptual knowledge, this might automatically prime pattern completion processes to retrieve information the individual knows, either from semantic memory or past trials, which is then used for encoding the newly presented information and leading to more general representations. This priming might reduce pattern separation processing in which detailed representations of the new information is formed. Although these different types of memory representations (general, detail) are both advantageous for particular tasks—general representations are useful for applying past experiences to new scenarios whereas detailed representations are information for recollecting a precise past—it is still unclear what task demands promotes access to each type of memory representation. Understanding these task demands is an exciting avenue for future research^42^.

Finally, some differences emerged across experiments when comparing the recognition memory for different object types. In the online Experiments 2 and 3, we found lower rates of false alarms for lures linked to initially learned objects, whereas in Experiment 1 the lower rates of false alarms were for lures linked to later learned objects. Future work is needed to fully understand whether this discrepancy is linked to differences in testing (online versus in person) or different strategies during the acquired equivalence task.

## Limitations and future directions

We acknowledge that our study has limitations. One limitation concerns the absence of an item-level analysis, which could show directly how the generalization of specific object-scene pairs influences subsequent recollection of individual objects. Our experimental design and data analyses were conducted at the group level, comparing the generalization performance and recall accuracy for conceptual and neutral pairs. To gain a better understanding of the potential trade-off between generalization and detailed recollection, as well as to extend our findings, future studies that examine this relationship at the item level are needed. Even with limitations, we consider that our study, and our focus on conceptual relatedness effects on generalization, has broad implications for understanding how semantic information supports memory processes. Here we show that prior knowledge can not only facilitate new learning and generalization but also in certain circumstances lead to a reduced ability in recalling detailed memories from our past. Understanding these circumstances is essential not only for scientific theory, but also to optimize learning in educational contexts^19^ and to help populations for whom episodic memory processing might be deficient, like in ageing^43^ and in clinical pathologies such as amnesia^44^ and depression^45^.

## Data Availability

All data and codes for all experiments are made available on the Open Science Framework project page, at https://osf.io/eyr7t/.
